# A randomised, multi-centre, prospective, double blind pilot-study to evaluate safety and efficacy of the non-absorbable Optilene^® ^Mesh Elastic versus the partly absorbable Ultrapro^® ^Mesh for incisional hernia repair

**DOI:** 10.1186/1471-2482-10-21

**Published:** 2010-07-12

**Authors:** Christoph Seiler, Petra Baumann, Peter Kienle, Andreas Kuthe, Jens Kuhlgatz, Rainer Engemann, Moritz v Frankenberg, Hanns-Peter Knaebel

**Affiliations:** 1 University of Heidelberg, Department of Surgery, Heidelberg, Germany; 2 Aesculap AG, Am Aesculap Platz, Tuttlingen, Germany; 3 University of Mannheim, Department of Surgery, Mannheim, Germany; 4 DRK-Hospital Clementinen, Hannover, Germany; 5 Albert-Schweitzer Hospital, Department of Surgery, Northeim, Germany; 6 Clinical Centre of Aschaffenburg, Department of Surgery, Aschaffenburg, Germany; 7 Hospital Salem, Department of Surgery, Heidelberg, Germany

## Abstract

**Background:**

Randomised controlled trials with a long term follow-up (3 to 10 years) have demonstrated that mesh repair is superior to suture closure of incisional hernia with lower recurrence rates (5 to 20% versus 20 to 63%). Yet, the ideal size and material of the mesh are not defined. So far, there are few prospective studies that evaluate the influence of the mesh texture on patient's satisfaction, recurrence and complication rate. The aim of this study is to evaluate, if a non-absorbable mesh (Optilene^® ^Mesh Elastic) will result in better health outcomes compared to a partly absorbable mesh (Ultrapro^® ^Mesh).

**Methods/Design:**

In this prospective, randomised, double blind study, eighty patients with incisional hernia after a midline laparotomy will be included. Primary objective of this study is to investigate differences in the physical functioning score from the SF-36 questionnaire 21 days after mesh insertion. Secondary objectives include the evaluation of the patients' daily activity, pain, wound complication and other surgical complications (hematomas, seromas), and safety within six months after intervention.

**Discussion:**

This study investigates mainly from the patient perspective differences between meshes for treatment of incisional hernias. Whether partly absorbable meshes improve quality of life better than non-absorbable meshes is unclear and therefore, this trial will generate further evidence for a better treatment of patients.

**Trial registration:**

NCT00646334

## Background

### Rationale

70.000 incisional hernia repairs were performed in Germany in 2006 [1]. Incisional hernias can cause serious complications such as incarceration or strangulation, resulting in substantial costs for further treatment (~ 128 Million €). Optimal treatment has not yet been defined [2,3].

Currently, the surgeon usually implants a mesh to reinforce the abdominal wall. The use of a mesh prosthesis for incisional hernia repair results in a lower recurrence rate than suture repair [4-11]. Creating a tension free repair with a mesh reduces the recurrence rate to 5-10%. Studies performed by Israelsson et al. in 2006 [12] and Kingsnorth et al. in 2004 [13] showed that the sublay technique seems to result in a lower recurrence rate (3-7%) compared to the onlay technique (12-19%). In order to achieve a sufficient reinforcement of the abdominal wall, the mesh should overlap the defect more than 5 cm in all directions [13-15]. Several meshes are available which differ in material, textile structure, pore size, weight, elasticity, tissue reaction, biocompatibility, and absorption [16-22]. Patients react differently to the mesh and the materials cause different complications such as seromas, chronic pain, and infections [14,15,19,23,24].

### Purpose

The aim of this study is to evaluate the safety and efficacy of the Optilene^® ^Mesh Elastic manufactured by B|Braun Aesculap compared to, the Ultrapro^® ^Mesh by Johnson&Johnson. Surgeons currently use both meshes to repair incisional hernias [25-27]. The two meshes have large pores based on polypropylene. Optilene^® ^Mesh Elastic is made of pure polypropylene and is not absorbable. Ultrapro^® ^is a partly absorbable mesh (polypropylene plus polyglecaprone, table 1).

**Table 1:** Comparison of the two meshes

Characteristics	Ultrapro	Optilene Mesh Elastic
Material	PP & PG (~ 1:1)	PP
Filament Structure	Monofil	Monofil
Construction	Knitted	Knitted
Weight after absorption of PG	65 g/m^2^ 28 g/m^2^	48 g/m^2^
Thickness	0,59 mm	0,55 mm
Pore Size	1.9 - 2.2 mm (min.- max.)	2.9 - 3.2 mm (min.-max.)
Absorption	partly absorbable	non-absorbable
Suture pull out test, lengthwise	33 N	33 N
Suture pull out test, crosswise	31 N	44 N

PP: Polypropylene, PG Polyglecaprone, N: Newton

## Methods/Design

### Study objectives

The primary objective of the study is to compare the physical functioning score from the SF-36 questionnaire 21 days after insertion of either an Optilene^® ^Mesh Elastic or an Ultrapro^® ^Mesh. Secondary objectives include the evaluation of the patients' daily activity, pain, wound assessment determined on several occasions during the observation time, the incidence of specific post-surgical complications and safety.

### Study design

**Figure 1:**
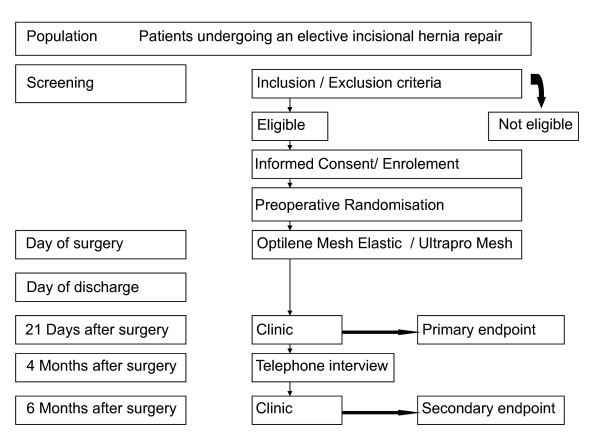
**Flow-chart of the trial**.

The study is a prospective, randomised, patient and observer blinded study. It is conducted in six centres in Germany. In total eighty patients with incisional hernia meeting the specific inclusion criteria will be randomised and followed for six months thereafter (table 2 and figure 1). Patients who prematurely terminate participation in the study will not be replaced.

**Table 2:** Tabular overview of the visits

	Visit 1 Pre- Surgery^1^	Visit 2 Surgery 0 Day	Visit 3 Release	Visit 4 Clinic 21 Days	Visit 5 Telephone 4 Months	Visit 6 Clinic 6 Months^2^
Patient information	X					

Informed Consent	X					

Demographics incl. employment status and home activities	X					

Body weight	X					X

Inclusion/Exclusion	X					

Medical history incl. history hernia	X					

Determination of potential risk factors	X					

Clinical examination	X					X

Concomitant medication	X	X^3^	X	X	X	X

General health status	X			X		X

SF-36	X			X		X

Daily activity questionnaire	X			X		X

Intra-operative details		X				

Adverse Events		X	X	X	X	X

Wound assessment			X	X		X

Seroma formation (sonography if indicated)			X	X		X

Pain score	X		X	X		X

Study termination						X

1) to be performed within 6 weeks before visit 2

2) or prematurely

3) concomitant medication except medication routinely given during a surgery and anaesthetic drug

### Study population

Female or male patients over 18 years old undergoing an elective repair for a midline incisional hernia are eligible for participation (table 3).

**Table 3:** Eligibility

Inclusion Criteria	Exclusion Criteria
Patient is female or male and ≥ 18 years old	Patient participates simultaneously in an investigational drug or medical device study
Female patients are incapable of pregnancy or must be using adequate contraception and are not in lactation	Patient has an acute incarcerated hernia Patient had a previous mesh repair at the same site
Patient has only a vertical aponeurotic incision	Enterotomy to be performed during hernia repair at Visit 2
Patient has an incisional hernia with a hernia size ≥ 3 cm	Patient is on anti-coagulation therapy
Patient is capable to understand and to follow the instructions	Patient is known or assessed to be non-compliant
Written informed consent is available	Patient must not get any additional surgical treatment at the same time (e. g. cholecystectomy)
Patient had no mesh implantation at the same site during a previous operation	Patient is immune incompetent (e. g. chemotherapy)

### Ethics and informed consent

The commercial regulatory authority Hannover gave its positive approval in February 2006. For the two centres in Heidelberg the Ethics Committee of the University of Heidelberg Medical School approved the final protocol on the 8^th ^Oktober 2007 and on 20^th ^November 2007. A central ethics approval was also obtain from the International Ethics Committee of Freiburg on the 4^th ^May 2009. Written informed consent will be obtained from all patients participating in the trial. The study is conducted in accordance with the principles of the Good Clinical Practice (GCP) guidelines, the Declaration of Helsinki, and the European Standard EN ISO 14155 Parts I and II (2003), "Clinical Investigation of Medical Devices for Human Subjects".

### Randomisation and blinding

Patients will be randomised by opening sealed, opaque envelopes containing the mesh to be implanted. The sponsor will prepare envelopes with a balanced distribution of meshes, according to the randomisation plan. The meshes will be assigned to patients in each centre in chronological order. Neither the patient nor the observer will have access to the documents indicating mesh distribution. The surgeon should not be the observer of outcomes in this clinical trial. Therefore, at least two different persons per centre are involved in this study, one who performs the surgery and the other one conducting the follow-up examinations. Together with the meshes the study centres receive emergency envelopes with the information of treatment allocation. The sponsor has to be contacted before breaking the code for a given patient. In case of opening the envelope, time, date, name of the person opening and the reason for opening the envelope are to be documented on that envelope and in the corresponding CRF.

### Intervention

In order to minimise bias and to assure parity in treatment for all patients, the following standardised procedures were implemented.

**Figure 2:**
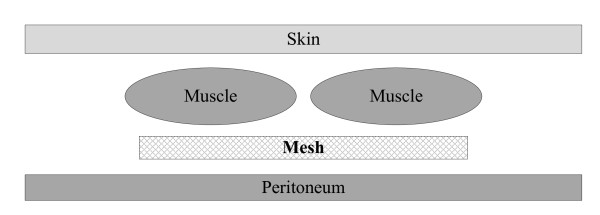
**Sublay technique for open incisional hernia repair**.

The operation is initiated with a vertical median incision. After classification of the hernia according to Schumpelick, a space is created between both posterior sheaths and the rectus muscle. The posterior fascia is closed using a running monofilament non-absorbable suture. The mesh is placed in sublay position between the posterior rectus sheath and the rectus muscle with an overlap of the defect of 5 cm in all directions (figure 2). Whereby the largest elasticity of the mesh is in vertical direction. The mesh is then fixed to the posterior fascia using a single knot technique every 3 cm with monofilament, non-absorbable suture material. The closure of the midline anterior rectus sheath is conducted with a continuous running technique using monofilament, non-absorbable sutures with a 4:1 ratio (suture length: incision length). Two Redon drains are placed close to the mesh. The skin is closed with tacks and an abdominal bandage is applied.

### Data collection and examinations

The investigator will collect data in a CRF about the patient and perform six examinations (table 2). CRF are paper-based and will be entered into a database by two persons independently applying plausibility checks. Queries raised during data base input will be clarified with the investigators.

### Questionnaires

The SF-36 Health Survey is a validated instrument to measure health status and patients are requested to complete the questionnaire before surgery, on day 21 after intervention and six months postoperatively.

### Documentation during and after surgery

During surgery, the investigator documents the size of the incision, the device and the material used for fixation, intra-operative complications, classification of the defect, and the size of the overlap of the mesh. The observer will record wound assessment, daily activity and pain as secondary endpoints (table 2).

### Safety aspects

The investigator has to document adverse events and serious adverse events on the appropriate form of the CRF which occur in the abdomen. Serious adverse events occurring during the study or within two weeks after discontinuation have to be reported to the sponsor within 24 hours of becoming aware of the event. It is the responsibility of the principal investigator at each centre to inform the local ethics committee of SAEs occurring at the centre according to local requirements.

### Sample size and statistical analysis

The primary efficacy endpoint is the change of SF 36 PCS between baseline and average of SF 36 PCS 21 days after intervention. The primary efficacy analysis will be conducted in the intention-to-treat population and applies a fixed effect linear model adjusting for age, BMI and SF 36 PCS before. Level of significance is set at 5% (two-sided).

Due to the lack of any empirical data for the primary endpoint in the population under investigation, there is substantial uncertainty with respect to overall rate and treatment effect to be expected. As a consequence, the assumptions to be made for sample size calculation are highly uncertain and therefore, the study is performed as a pilot randomised trial with 80 patients.

Secondary endpoints are level of function and daily activity, seroma formation, wound assessment, neuralgias, time to return to work and to normal activities, the patient's rating of pain, analgesic consumption and other SF-36 scores during 6 months after surgery. These data will be analysed descriptively. No confirmatory statistical testing will be done with regard to secondary endpoints. Details of the analysis of secondary outcome parameters will be documented before database lock in the analysis-plan. The safety assessments, including adverse events and serious adverse events, will be analysed descriptively.

### Trial organization, coordination and registration

This study is initiated and sponsored by B|Braun Aesculap. Aesculap AG conducts it in cooperation with the CRO Dr. med. Lenhard&Partner GmbH. The CRO is responsible for monitoring, biostatistics and database. Aesculap AG is responsible for the project management. The sponsor supplies the participating trial centres with the meshes used in the trial. Aesculap AG is responsible for the registration (Identifier Number NCT 00646334, http://www.clinicaltrials.gov) and all trial related meetings.

### Monitoring

Data documentation and case report forms (CRF) will be reviewed for accuracy and completeness during on-site monitoring visits and at the sponsor's site. The first monitoring visit after the study initiation will be made as soon as the enrolment of patients has begun. On these visits, the monitor will perform source data verification, i.e. compare the data entered in the CRFs with the hospital records. The trial centres may be visited either by representatives of the sponsor or the local authorities to perform an audit.

### Current status

The first investigator meeting was held on 21^st ^December 2005 in Tuttlingen, Germany. The study protocol for this trial was completed on 19^th ^January 2006. In February 2006, following completion of contracts the first three centres (Hannover, Aschaffenburg, Northeim) were initiated, the first patient was recruited in July 2006. Due to slow accrual of patients three other centres (University of Heidelberg, University of Mannheim, Salem Hospital in Heidelberg) were initiated in December 2007. It is expected that the last patient will be randomised in November 2009. The study is estimated to be completed in June 2010.

## Discussion

Incisional hernia is a common complication after abdominal surgery with a reported incidence between 11 and 20 percent [6,8,10,28]. Such hernias can cause serious complications such as strangulation or incarceration [2,3]. Many techniques are currently in use to repair incisional hernias. Primary suture repair has been widely used, but results in a high recurrence rate between 24% and 54% [5,10,29,30]. With the development of new synthetic materials the use of prosthetic meshes has gained popularity in the treatment of incisional and ventral hernias [31]. The mesh facilitates closure, minimizes tension on the suture line, and assures high wound strength [32,33]. The use of prosthetic mesh is associated with a lower incidence of hernia recurrence, ranging from 2 to 36 percent [5,10,11]. A prospective, long-term, comparative study showed that, for both small and large incisional hernias, mesh repair was superior to suture repair in regards to recurrence [8]. In addition the incidence and intensity of abdominal pain were also lower after mesh repair than after suture repair.

Several trials have been performed in order to find the optimal mesh [19,24,34-37] and the ideal technique for implantation [13,38]. The onlay and the sublay technique are used in open mesh repair [12,13,38]. Both techniques give good results but the sublay technique seems superior in regard to complications and recurrence rate. The inlay technique is nowadays rather rarely used [9,12,38]. The size of the prosthesis is also important for the recurrence rate of incisional hernias [24,39-41]. The mesh should coverlap the defect more than 5 cm in all directions from the margin of the hernia, in order to achieve a sufficient reinforcement of the abdominal wall [13-15,24,41].

The manifold available meshes differ from each other in their material, in their textile structure and in their tissue reaction and absorption. Evaluation of the different meshes for incisional hernia repair is of special interest because they are different in their biocompatibility and complication rate. Pain, seroma and persisting infection are known mesh-related complications [15]. Most studies showed a high incidence of seroma formation after mesh repair [5,8,10,42]. But with conservative treatment most of these eventually resolve. The inflammatory activity of the mesh mainly depends on the amount of material and its textile structure [35,43,44]. In accordance, the majority of these problems are associated with small pore-sized, heavy-weight, meshes [15]. In some patients, an excessive shrinkage of these meshes cause considerable complaints and even require a mesh change [15]. To overcome this problem, another form of mesh was introduced the large pore-sized, light-weight mesh. They rarely cause severe mesh-related problems, due to their reduced amount of polymer [19,24]. With these materials, patients report less pain, less mesh awareness and show less symptoms such as a "stiff abdomen" [19,23,24].

Partly absorbable meshes have also been compared with non-absorbable heavy-weight large pore-sized meshes [19,24]. No difference in the incidence of wound infections and the rate and the volume of seroma were found [19,24]. But these studies did not analyse the role of the absorbable and the non-absorbable part in causing complications [19]. Currently most surgeons favour large pore-sized, light-weight, elastic, monofilament polypropylene meshes in the sublay position for reinforcement of the abdominal wall [14,19,23].

There are only few prospective studies that evaluate the influence of the mesh texture on patient's Quality of Life. So far no randomised controlled trial, which evaluates if the absorbable part of a mesh increases the rate of wound infections, pain, patients discomfort, and other complications after mesh implantation has been published. It remains unclear whether the application of partially absorbable components might contribute to improvement of the biocompatibility of polypropylene meshes and whether such improvement would decrease the incidence of wound infections or other complications. Therefore, this study was designed, focusing on patient related outcomes.

## Abbreviations

AE: Adverse Event; BMI: Body Mass Index; CRF: Case Report Form; CRO: Clinical Research Organisation; GCP: Good Clinical Practice; SAE: Serious Adverse Event; SF 36 PCS: SF-36 Physical Component Summary

## Competing interests

Aesculap AG, Germany, sponsors this study and its publication.

## Authors' contributions

PB and HPK (B|Braun Aesculap, Tuttlingen, Germany) managed and conducted the trial in co-operation with Dr. med Lenhardt&Partner GmbH. CS wrote the manuscript together with PB and HPK. All authors have read and approved this manuscript.

## Pre-publication history

The pre-publication history for this paper can be accessed here:

http://www.biomedcentral.com/1471-2482/10/21/prepub

## References

[B1] Conze J Junge K Klinge U Krones C Rosch R Schumpelick V Evidenzbasierte laparoskopische Chirurgie - Narbenhernien Viszeralchirurgie 2006 41 246 252 10.1055/s-2006-942141

[B2] Read RC Yoder G Recent trends in the management of incisional herniation Arch Surg 1989 124 485 488 2649047 10.1001/archsurg.1989.01410040095022

[B3] Manninen MJ Lavonius M Perhoniemi VJ Results of incisional hernia repair. A retrospective study of 172 unselected hernioplasties Eur J Surg 1991 157 29 31 1675878

[B4] Flum DR Horvath K Koepsell T Have outcomes of incisional hernia repair improved with time? A population-based analysis Ann Surg 2003 237 129 135 10.1097/00000658-200301000-00018 12496540 PMC1513979

[B5] Luijendijk RW Hop WC Van Den Tol MP De Lange DC Braaksma MM Ijzemans JN A comparison of suture repair with mesh repair for incisional hernia N Engl J Med 2000 343 392 398 10.1056/NEJM200008103430603 10933738

[B6] Al-Salamah SM Hussain MI Khalid K Al-Akeely MH Suture versus mesh repair for incisional hernia Saudi Med J 2006 27 652 656 16680255

[B7] Sauerland S Schmedt CG Lein S Leibl BJ Bittner R Primary incisional hernia repair with or without polypropylene mesh: a report on 384 patients with 5-year follow-up Langenbecks Arch Surg 2005 390 408 412 10.1007/s00423-005-0567-2 16028087

[B8] Burger JW Luijendijk RW Hop WC Halm JA Verdaasdonk EG Jeekel J Long-term follow-up of a randomized controlled trial of suture versus mesh repair of incisional hernia Ann Surg 2004 240 578 583 15383785 10.1097/01.sla.0000141193.08524.e7 PMC1356459

[B9] Langer C Liersch T Kley C Flosman M Süss M Siemer A Becker H [Twenty -five years of experience in incisonal hernia surgery. A comparative retrospective study of 432 incisonal hernia repairs] Chirurg 2003 74 638 645 10.1007/s00104-002-0594-2 12883791

[B10] Korenkov M Sauerland S Arndt M Bogradi L Neugebauer EAM Troidl H Randomized clinical trial of suture repair, polypropylene mesh or autodermal hernioplasty for incisional hernia Br J Surg 2002 89 50 56 10.1046/j.0007-1323.2001.01974.x 11851663

[B11] Liakakos T Karanikas I Panagiotidis H Dendrinos S Use of Marlex mesh in the repair of recurrent incisional hernia Br J Surg 1994 81 248 249 10.1002/bjs.1800810230 7710471

[B12] Israelsson LA Smedberg S Montgomery A Norgin P Spangen L Incisional hernia repair in Sweden 2002 Hernia 2006 10 258 261 10.1007/s10029-006-0084-4 16554979

[B13] Kingsnorth AN Sivarajasingham N Wong S Butler M Open mesh repair of incisonal hernia with significant loss of domain Ann R Coll Surg Engl 2004 86 363 366 10.1308/147870804236 15333175 PMC1964255

[B14] Conze J Kingsnorth AN Flament JB Simmermacher R Arlt G Langer C Schippers E Hartley M Schumpelick V Randomized clinical trial comparing lightweight composite mesh with polyester or polypropylene mesh for incisional hernia repair Br J Surg 2005 92 1488 1493 10.1002/bjs.5208 16308855

[B15] Conze J Krones CJ Schumpelick V Klinge U Incisional hernia: challenge of re-operations after mesh repair Langenbecks Arch Surg 2007 392 453 457 10.1007/s00423-006-0065-1 16951970

[B16] Schug-Paß C Tamme C Sommerer F Lippert H Köckerling F A lightweight, partially absorbable mesh (Ultrapro) for endoscopic hernia repair: experimental biocompatibility results obtained in a porcine model Surg Endosc 2007 22 1100 1106 10.1007/s00464-007-9585-1 17963002

[B17] Scheidbach H Tannapfel A Schmidt U Lippert H Köckerling F Influence of titanium coating on the biocompatibility of a heavyweight polypropylene mesh Eur Surg Res 2004 36 313 317 10.1159/000079917 15359095

[B18] Scheidbach H Tamme C Tannapfel A Lippert H Köckerling F In vivo studies comparing the biocompatibility of various polypropylene meshes and their handling properties during endoscopic total extraperitoneal (TEP) patchplasty Surg Endosc 2004 18 211 220 10.1007/s00464-003-8113-1 14691711

[B19] Welty G Klinge U Klosterhalfen B Kasperk R Schumpelick V Functional impairment and complaints following incisional hernia repair with different polypropylene meshes Hernia 2001 5 142 147 10.1007/s100290100017 11759800

[B20] Junge K Rosch R Klinge U Saklak M Klosterhalfen B Peiper C Schumpelick V Titanium coating of a polypropylene mesh for hernia repair: effect on biocompatibility Hernia 2005 9 115 119 10.1007/s10029-004-0292-8 15583967

[B21] Junge K Rosch R Krones CJ Klinge U Mertens PR Lynen P Schumpelick V Klosterhalfen B Influence of polyglecaprone 25 (Monocryl) supplementation on biocompatibility of a polypropylene mesh for hernia repair Hernia 2005 9 212 217 10.1007/s10029-004-0315-5 15703859

[B22] Schug Paß C Tamme C Köckerling F A lightweight polypropylene mesh (TiMesh) for laparoscopic intraperitoneal repair of abdominal wall hernias comparison of biocompatibility with the Dual mesh in an experimental study using the porcine model Surg Endosc 2006 20 402 209 10.1007/s00464-004-8277-3 16432656

[B23] Schmidbauer S Ladurner R Hallfeldt KK Mussack T Heavy-weight versus low-weight polypropylene meshes for open sublay mesh repair of incisional hernia Eur J Med Res 2005 10 247 253 16033714

[B24] Schumpelick V Klosterhalfen B Müller M Klinge U Minimized polypropylene mesh for preperitoneal net plasty (PNP) of incisional hernias Chirurg 1999 70 422 430 10.1007/s001040050666 10354839

[B25] Benhidjeb T Bärlehner E Anders S Laparoskopische Narbenhernien-Reparation- Muss das Netz für die intraperitoneale Onlay-Mesh-technik besondere Eigenschaften haben? Chir Gastroenterol 2003 19 suppl 2 16 22 10.1159/000076183

[B26] Rosen HR Gyasi A Retromuskuläre Kunststoff-Netz-Implantation von Narbenhernien Chir Gastroenterol 2003 19 suppl 2 39 45 10.1159/000076187

[B27] Rosch R Junge K Stumpf M Klinge U Schumpelick V Klosterhalfen B Welche Anforderungen sollte ein ideales Netz erfüllen? Chir Gastroenterol 2003 19 suppl 2 7 11 10.1159/000076181

[B28] Höer J Lawong G Klinge U Schumpelick V [Factors influencing the development of incisonal hernia. A retrospective study of 2,983 laparotomy patients over a period of 10 years] Chirurg 2002 73 474 480 10.1007/s00104-002-0425-5 12089832

[B29] Luijendijk RW Lemmen MH Hop WC Wereldsma JC Incisional hernia recurrence following "vest-over-pants" or ventral mayo repair of primary hernias of the midline World J Surg 1997 21 62 65 10.1007/s002689900194 8943179

[B30] Luijendijk RW "Incisional hernia": risk factors, prevention, and repair (PhD.thesis) 2000 Rotterdam, The Netherlands: Erasmus University Rotterdam

[B31] Itani KMF Neumayer L Reda D Kim L Anthony T Repair of ventral incisional hernia: the design of a randomized trial to compare open and laparoscopic surgical techniques Am J Surg 2004 188 22 29 10.1016/j.amjsurg.2004.09.006 15610889

[B32] Vrijland WW Jeekel J Steyerberg EW Den Hoed PT Bonjer HT Intraperitoneal polypropylene mesh repair of incisional hernia is not associated with enterocutaneous fistula Br J Surg 2000 87 348 352 10.1046/j.1365-2168.2000.01364.x 10718806

[B33] Christoforoni PM Kim YB Preys Z Lay RY Montz FJ Adhesion formations after incisional hernia repair: a randomized porcine trial Am Surg 1996 62 935 938 8895716

[B34] Weyhe D Belyaev O Müller C Meurer K Bauer KH Papapostolou G Uhl W Improving outcomes in hernia repair by the use of light meshes - a comparison of different implant constructions based on a critical appraisal of the literature World J Surg 2007 31 234 244 10.1007/s00268-006-0123-4 17180568

[B35] Klosterhalfen B Klinge U Schumpelick V Functional and morphological evaluation of different polypropylene-mesh modifications for abdominal wall repair Biomaterials 1998 19 2235 2246 10.1016/S0142-9612(98)00115-X 9884036

[B36] Klosterhalfen B Junge K Klinge U The lightweight and large porous mesh concept for hernia repair Expert Rev Med Device 2005 2 103 117 10.1586/17434440.2.1.103 16293033

[B37] Schumpelick V Klinge U Junge K Stumpf M Incisional abdominal hernia: the open mesh repair Langenbecks Arch Surg 2004 389 1 5 10.1007/s00423-003-0352-z 14745557

[B38] Langer C Neufang T Kley C Schönig KH Becker H [Standardized sublay technique in polypropylene mesh repair of incisional hernia] Chirurg 2001 72 953 7 10.1007/s001040170095 11554142

[B39] De Vries Reilingh TS van Geldere D Langenhorst B de Jong D van der Wilt GJ van Goor H Bleichrodt RP Repair of large midline incisional hernias with polypropylene mesh: comparison of three operative techniques Hernia 2004 8 56 59 10.1007/s10029-003-0170-9 14586775

[B40] Ladurner R Trupka A Schmidbauer S Hallfeldt K The use of an underlay polypropylene mesh in complicated incisional hernias: sucessful French surgical technique Minerva Chir 2001 56 111 117 11283488

[B41] Schumpelick V Klinge U Junge K Stumpf M Incisional abdominal hernia: the open mesh repair Langenbecks Arch Surg 2004 389 1 5 10.1007/s00423-003-0352-z 14745557

[B42] Machairas A Misiakos EP Liakakos T Karatzas G Incisional hernioplasty with extraperitoneal onlay polyester mesh Am Surg 2004 70 726 729 15328809

[B43] Klosterhalfen B Klinge U Hermanns B Schumpelick V Pathology of traditional surgical nets for hernia repair after longterm implantation in humans Chirurg 2000 71 43 51 10663001 10.1007/s001040050007

[B44] Klosterhalfen B Klinge U Hermanns B Schumpelick V Pathology of traditional surgical nets for hernia repair after longterm implantation in humans Chirurg 2000 71 43 51 10663001 10.1007/s001040050007

